# The Effect of Music Stimulation on Resting-State Brain Functional Networks Following Exhaustive Endurance Exercise: An EEG Study

**DOI:** 10.3390/brainsci16030258

**Published:** 2026-02-25

**Authors:** Jing Fan, Bohan Li, Fujie Liu, Fanghao Jiao, Aiping Chi, Shuqi Yao

**Affiliations:** 1 College of Education and Public Service, Shaanxi Vocational & Technical College, Xi’an 710100, China; 2 School of Sports, Shaanxi Normal University, Xi’an 710119, China

**Keywords:** music, endurance exercise, network connectivity, phase locking value

## Abstract

**Objective:** The purpose of this research is to examine how motivational music immediately impacts the brain’s functional connectivity patterns in male athletes following a single session of intense endurance exercise, utilizing resting-state electroencephalography (EEG) and brain network analysis methods. **Methods:** The study involved 34 healthy male athletes who were tasked with performing incremental cycling exercises until exhaustion, both with and without music. Their resting-state EEG was recorded before and after the exercise. Brain functional networks were analyzed in the theta, alpha, and beta frequency bands based on changes in phase locking value (PLV). Specifically, the study examined the central executive network (CEN), default mode network (DMN), salience network (SN), sensorimotor network (SMN), and dorsal attention network (DAN), assessing their topological properties using graph theory methods. **Results:** Music significantly prolonged the time to exhaustion. Across frequency bands, the music condition exhibited higher global and local efficiency compared with the no-music condition. Following exhaustion without music, beta-band connectivity significantly increased, suggesting compensatory hyper-synchronization under fatigue. In contrast, music led to reduced alpha- and beta-band global connectivity post-exercise, accompanied by selective strengthening of functionally relevant couplings, particularly between SMN and CEN, and enhanced DAN–DMN coordination. Additionally, music prevented maladaptive connectivity shifts observed under fatigue, including excessive SN–CEN coupling. **Conclusions:** Exhaustive exercise without music induces widespread beta-band hyper-connectivity, reflecting increased neural cost under central fatigue. Music, however, promotes a more efficient and selectively integrated network configuration, supporting the neural efficiency hypothesis. These findings provide neurophysiological evidence that music optimizes large-scale brain network organization under physical stress, thereby contributing to enhanced endurance performance.

## 1. Introduction

Endurance exercise, as a form of prolonged, moderate-intensity physical activity, plays an irreplaceable role in promoting cardiovascular health, improving metabolic function, and delaying the aging process [1]. The biochemical adaptation of skeletal muscles forms the basis for endurance enhancement, with maximal oxygen uptake, lactate threshold, and exercise economy collectively determining endurance performance [2,3]. However, when exercise reaches exhaustion, factors limiting performance stem not only from peripheral muscle energy depletion or metabolic byproduct accumulation, but also from the crucial role of central nervous system (CNS) fatigue [4]. Therefore, exploring interventions that can effectively regulate CNS status and delay central fatigue is of significant importance for enhancing athletic performance and improving the exercise experience.

Music, as a non-pharmacological, non-invasive, and easily implementable intervention, has been widely proven to have ergogenic effects in the field of exercise [5,6]. Numerous studies have shown that listening to self-selected or motivational music during exercise can effectively enhance endurance, delay fatigue, reduce rated perceived exertion (RPE), and improve mood [7]. These effects have been validated not only in healthy individuals and athletes but also demonstrate significant potential in the elderly [8] and clinical rehabilitation populations [9]. However, the neural mechanisms through which music exerts these effects remain unclear. While traditional explanations such as attention diversion, synchronization of movement rhythms, or modulation of emotional arousal have some merit, they have not delved deeply into the brain’s functional network level to reveal how music systematically influences the brain’s working patterns.

In recent years, the advancement of neuroimaging techniques, particularly electroencephalography (EEG), has made it possible to explore brain activity in the field of sports science. EEG, with its high temporal resolution, captures changes in brain electrical activity in milliseconds, making it ideal for studying brain responses during dynamic processes like movement [10]. The introduction of dry-electrode EEG systems has greatly simplified preparation procedures, making data collection outside the laboratory more convenient and further promoting its application in sports training settings [11]. By combining graph theory and other brain network analysis methods, researchers can examine how different brain regions cooperate at a macroscopic level, forming functional networks, and how the topological properties and connectivity patterns of these networks change with task conditions such as fatigue or music intervention [12].

Research has begun to utilize EEG to investigate the impact of exercise on brain function. For instance, studies have shown that exhaustive exercise or sleep deprivation can alter the functional connectivity between the brain cortex and muscles, affecting performance [13,14]. Simultaneously, the influence of music on brain activity is gaining attention, with techniques like music neurofeedback being used to regulate emotions and cognition [15,16]. However, the combination of these two aspects, systematically studying how music modulates the brain functional network after exhaustive endurance exercise, remains relatively rare.

This study aims to investigate the immediate effects of motivational music on the resting-state brain functional connectivity of male athletes after exhaustive endurance exercise. The study combines high temporal resolution EEG technology with brain network analysis methods. The hypothesis is that (1) listening to motivational music will significantly enhance endurance performance compared to the no-music condition; (2) exhaustive exercise will cause significant reorganization of the brain’s functional networks to cope with physiological and psychological stress; (3) music intervention will modulate this reorganization process in a unique way, potentially promoting a more efficient and economical neural working mode, thus providing direct neurophysiological evidence for the enhancing effects of music.

## 2. Materials and Methods

### 2.1. Participants

The study conducted a priori power analysis using G*Power 3.1 software (version 3.1, Heinrich-Heine-Universität Düsseldorf, Düsseldorf, Germany). With a significance level (α) set at 0.05, a power (1-β) of 0.80, and an expected effect size of 0.25 [17], the analysis indicated a need for 27 participants. Ultimately, 34 male college students majoring in sports (mean age: 23.5 ± 2.31) were recruited for the experiment. All participants met the following inclusion criteria: (1) good physical health without a history of genetic diseases, brain injuries, cardiovascular diseases, or mental/neurological disorders; (2) normal uncorrected or corrected vision without color vision deficiencies; (3) no recent physical injuries or excessive fatigue; (4) right-handed; (5) regular exercisers based on the International Physical Activity Questionnaire assessment; (6) complied with pre-experiment requirements: no vigorous exercise, alcohol, caffeine, or medication intake within 24 h before testing, and stable emotional state; (7) signed informed consent indicating full understanding of the experimental procedures, voluntary participation, and compensation upon completion.

### 2.2. Endurance Exercise Model

The study utilized the Ergoline brand (model Ergoline 800, Ergoline GmbH, Bitz, Germany) power cycle as experimental equipment to implement an incremental load to exhaustion exercise protocol [18]. A single bout of exhaustive endurance exercise was performed using an incremental load exercise mode [19,20]. Participants cycled on the power cycle with incremental loads, each stage consisting of a set load, cadence (maintained at 60 rpm throughout), and time (each stage/3 min), with higher stages indicating higher loads. Participants started at an initial load of 50 watts (W) and increased by 50 W every 3 min, with the fourth stage having no load increment or time limit, until voluntary exhaustion [4]. Prior to the formal test, participants completed a 3 min warm-up with a load set at 25 W. During the experiment, participants’ heart rate changes were monitored using a physical activity monitor (GT9-X, ActiGraph LLC, Pensacola, FL, USA) worn on the left hand. Additionally, at the end of each stage, participants were asked to rate their perception of effort on the RPE scale [21] for each stage of the power cycle, and the responses were recorded.

The termination criteria for the exhaustive exercise protocol are met when the participant simultaneously meets three out of the following four criteria: (1) subjective physical state: experiencing difficulty breathing accompanied by profuse sweating; (2) cycling cadence: despite verbal encouragement, unable to maintain a cadence of 60 rpm and consistently falling below this standard for more than 10 s; (3) heart rate indicator: reaching or approaching their personal maximum heart rate (calculated using the formula: 220 − age); (4) subjective fatigue perception: RPE level reaching 18 points or above, and despite repeated encouragement from the researchers, unable to continue exercising.

### 2.3. Music Selection

To ensure that the music materials used in the experiment effectively evoke the desired motivational emotions, standardized procedures were employed for music selection. All selection processes took place in a well-soundproofed laboratory to eliminate environmental noise interference. Participants were instructed to listen to “ppy” music from a library of Chinese emotional music materials in a quiet environment and immediately fill out the Brunel Music Rating Inventory-2 (BMRI-2) after each music piece ended [22]. Subsequently, the music corresponding to each participant’s BMRI-2 score was determined, with tracks scoring ≥ 36 considered for inclusion to ensure that the music provided participants with effective external motivation and emotional support during exercise. During the exhaustive cycling task, the selected music track was played continuously in a loop through headphones to ensure consistent auditory stimulation throughout the exercise period. The volume was kept constant at a comfortable (75 dB) and clearly audible level across all participants to minimize variability due to differences in sound intensity. The selected music tracks primarily ranged between approximately 120 and 160 beats per minute, and all tracks were instrumental (without lyrics). In the no-music condition, participants wore the same headphones without any auditory stimulation to control for potential equipment-related effects. The music was played using Beats Fit Pro noise-canceling headphones(Beats Electronics LLC, Culver City, CA, USA).

### 2.4. Acquisition and Processing of Electrical Signals

**Figure 1:**
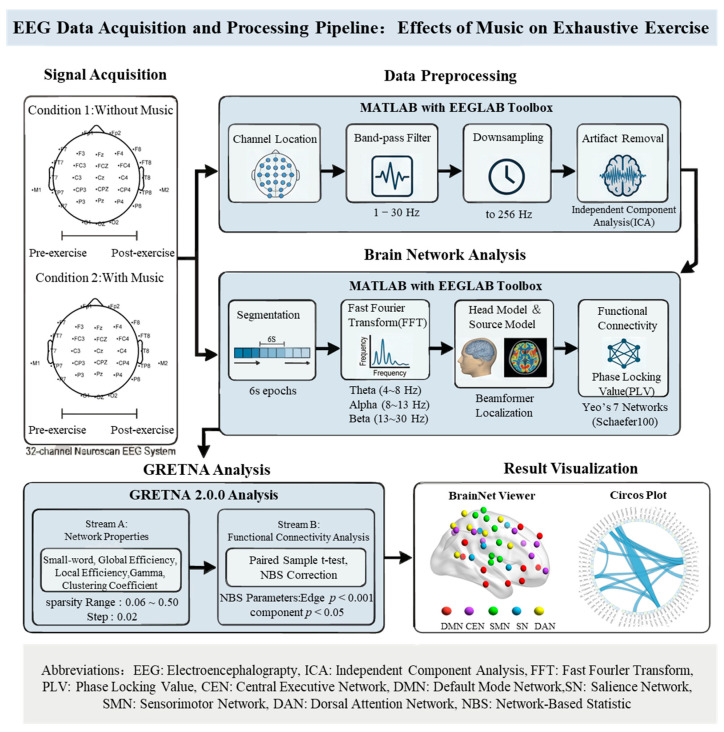
Flowchart of the processing and analysis of EEG data.

A randomized within-subject crossover design was employed. Each participant completed both the music and no-music conditions in separate sessions, with the order counterbalanced across participants. To prevent fatigue carryover effects, a washout period of at least 72 h was implemented between sessions. All sessions were conducted at the same time of day (±1 h) for each participant to control for circadian effects. The signal was collected using a 32-channel Neuroscan EEG system (Brain Vision Recorder, Neuroscan, Charlotte, NC, USA), with electrodes placed according to the international 10-10 system [23], and scalp impedance was kept below 10 kΩ. EEG data were collected both before and immediately after exhaustion, during a 5 min resting state. Participants were instructed to clean their heads before the data collection and to keep their eyes closed and remain still to avoid eye movements, swallowing, or other actions during the recording process. EEG data were acquired immediately following the exhaustive exercise protocol. Participants remained seated, and the EEG recording commenced within 30 s after exercise termination. During this period, participants were instructed to maintain a standardized resting posture (comfortably seated with back supported and hands resting on thighs) and to breathe in a relaxed, steady manner. The processing flow of the EEG data is outlined in Figure 1.

**Figure 2:**
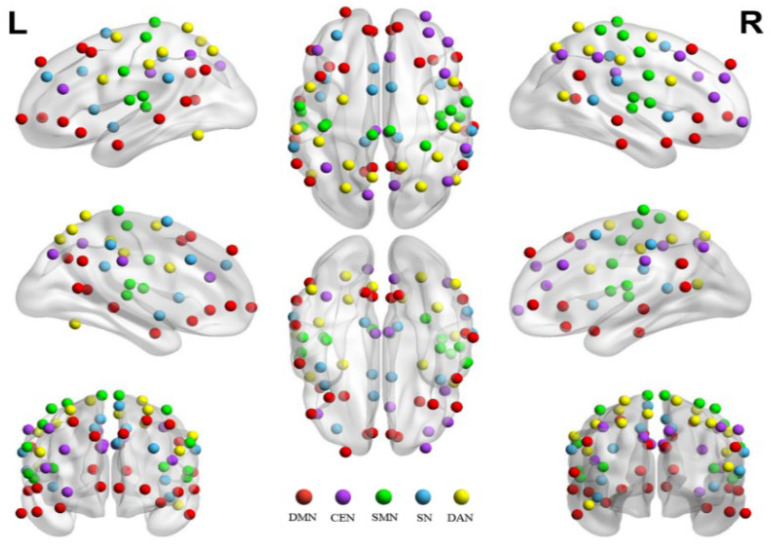
Spatial distribution of brain networks of interest. Note: L and R indicate left and right viewing perspectives, respectively.

Import the collected EEG data into MATLAB software (Version R2024b, USA). EEG data were preprocessed using the EEGLAB toolbox (version 2025.1.0). The processing pipeline included channel localization, bandpass filtering (1–30 Hz), and downsampling to 256 Hz. Independent component analysis (ICA) was then applied to remove artifacts, including those related to eye blinks, eye movements, head movements, and cardiac activity [24]. Artifactual components were identified using ICLabel, with classifications subsequently confirmed through visual inspection. These components were then excluded from the data to yield a clean EEG dataset. Identical preprocessing procedures were applied across both conditions. Artifact removal using ICA was performed blind to condition, and epochs containing excessive noise or movement artifacts were excluded using identical rejection criteria. Segment the preprocessed EEG data into 6 s intervals, then load the FieldTrip toolbox [25] in MATLAB. Process the EEG data with procedures including fast Fourier transform, loading head and source models, setting up connectivity metrics calculation, and loading brain network templates. Key parameters include selecting frequency bands of interest such as theta (4–8 Hz), alpha (8–14 Hz), and beta (14–30 Hz) [26]. Choose the Beamformer method for brain region localization; Yeo’s 7 major brain networks, Schaefer100 template for brain network template [27]. Select the central executive network (CEN), default mode network (DMN), salience network (SN), sensorimotor network (SMN), and dorsal attention network (DAN) as regions of interest (78 brain regions in total) as part of the brain networks of interest (Figure 2). Use the Phase Locking Value (PLV) as the connectivity metric to assess the phase synchronization between neural oscillations of two brain regions (X and Y).

### 2.5. Connectivity Analysis of Brain Functional Networks

Import the PLV data of the 78 brain regions into GRETNA 2.0.0 [28] for the following analyses: (1) analysis of brain network properties: calculate small-world properties [6] within a sparsity range of 0.06 to 0.50 with a step size of 0.02 (total of 23 sparsity levels). This range was selected based on prior EEG network studies and graph theoretical criteria to ensure that the resulting networks remained fully connected while minimizing the inclusion of weak or potentially spurious connections. For each sparsity level, weighted PLV matrices were thresholded to retain the strongest connections while maintaining equal network density across participants and conditions. The thresholded matrices were then converted into binary adjacency matrices and used to compute graph theoretical metrics, evaluate global network metrics, including global efficiency (Eglob), local efficiency (Eloc), global clustering coefficient (Cp), and normalized clustering coefficient (Gamma). If sigma value > 1, the formulas for the network metrics are as follows:

(a) Gamma (γ): the normalized clustering coefficient γ reflects the degree of local clustering or functional segregation of the network. It is calculated as

$$\gamma =\frac{C_{p}}{C_{random}}$$

where $C_{p}$ is the clustering coefficient of the real network, and $C_{random}$ is the mean clustering coefficient of 1000 matched random networks. A value of γ > 1 indicates that the network exhibits stronger local clustering than random networks.

(b) Normalized characteristic path length (Lambda, λ): the normalized characteristic path length λ reflects the global information integration efficiency of the network. It is calculated as

$$\lambda =\frac{L_{p}}{L_{random}}$$

where $L_{p}$ is the characteristic path length of the real network, and $L_{random}$ is the mean characteristic path length of the matched random networks.

(c) Sigma (σ): the small-worldness index σ quantifies whether the network simultaneously exhibits high local clustering and short global path lengths. When σ > 1, the network is considered to exhibit small-world properties. It is calculated as

$$\sigma =\frac{\gamma }{\lambda }$$

(d) Cp: the clustering coefficient $C_{p}$ is defined as the average clustering coefficient across all nodes in the network, reflecting the overall local interconnectedness of the network. It is calculated as

$$C_{p}=\frac{1}{N}\sum_{i\in G}\frac{E_{i}}{D_{i}(D_{i}-1)/2}$$

where N is the number of nodes in the network, $E_{i}$ is the number of existing edges among the neighbors of node i, and $D_{i}$ is the degree of node i.

(e) Eglob: global efficiency is the inverse of the characteristic path length and reflects the overall capacity for parallel information transfer and integration in the network. It is particularly suitable for disconnected networks. Higher global efficiency indicates more efficient information exchange across the network. It is calculated as

$$E_{glob}=\frac{1}{N\left(N-1\right)}\sum_{i\neq j}\frac{1}{L_{ij}}$$

where $L_{ij}$ is the shortest path length between nodes i and j.

(f) Eloc: local efficiency measures the fault tolerance of the network and reflects the efficiency of information transfer among the neighbors of each node. It is closely related to the clustering coefficient and reflects the functional segregation of the network [29]. It is calculated as

$$E_{loc}=\frac{1}{N_{G_{i}}(N_{G_{i}}-1)}\sum_{j\neq k\in G_{i}}\frac{1}{L_{i,k}}$$

(2) Analysis of functional connectivity between brain regions: use paired sample *t*-tests for inter-group comparisons. Apply the Network-Based Statistic (NBS) method for multiple comparison correction (NBS correction parameters: Edge *p* < 0.001, component *p* < 0.05, 2000 permutations). The total average functional connectivity strength was calculated as the mean PLV value across all pairwise node connections in the network, excluding self-connections. This measure reflects the overall level of phase synchronization across the brain network and provides a global index of functional integration. Visualize brain region connectivity maps with the BrainNetViewer toolkit (version 1.7) [30] and the circos toolkit [31] for significantly different connections. (3) Analysis of network connection strength: Normalize connection strength coefficients using the Min-Max method. Calculate functional connection strengths within and between the CEN, DMN, SN, SMN, and DAN. All connectivity analyses and visualizations were based on group-level statistical comparisons across all 34 participants. Although source-level reconstruction and beamforming reduce volume conduction effects, PLV may still be influenced by residual signal leakage or zero-lag coupling. Future studies may complement PLV with connectivity measures less sensitive to instantaneous phase synchronization, such as imaginary coherence or weighted phase lag index (wPLI), to enhance robustness.

### 2.6. Mathematical Statistics

Perform statistical analysis on the data using SPSS (Version 27.0; SPSS, Inc., Chicago, IL, USA) and assess the normal distribution of the data using the Shapiro–Wilk test. Present normally distributed data in the form of mean ± standard deviation. For global brain network property metrics, a 2 × 2 repeated-measures ANOVA was conducted with Condition (without music vs. with music) and Time (pre-exercise vs. post-exercise) as within-subject factors to examine main effects and interaction effects. Since the within- and between-network functional connectivity strength in this study are essentially ranked variables (ranks) and do not conform to the assumption of continuous normal distribution, non-parametric tests were therefore applied. For the four conditions (pre-exercise without music, pre-exercise with music, post-exercise without music, and post-exercise with music), the Friedman test was used for overall comparison, and the Dunn’s multiple comparisons test was employed for post hoc pairwise comparisons, with the Bonferroni method applied to correct the significance level. The significance level is set at *p* < 0.05 for significant differences and *p* < 0.01 for highly significant differences.

## 3. Results

### 3.1. Effects of Music on Endurance Exercise Performance

All participants successfully completed two incremental load endurance exercises without any dropouts or adverse reactions. The evaluation indicators of the exercise model are shown in Table 1. Participants reached a state of physiological exhaustion at the end of the exercise: (1) at exercise termination, all participants’ heart rates exceeded 180 beats/min; (2) subjective fatigue sensation: participants reported a subjective fatigue sensation close to the maximum score of 20 on the scale, indicating an “extreme fatigue” level, suggesting that participants subjectively could no longer sustain the current exercise intensity or found it extremely challenging; (3) participants experienced breathing difficulties at the end of the exercise; (4) participants were unable to maintain a cycling pedal speed of 60 rpm at the end of the exercise.

Based on multiple indicators such as heart rate, RPE, and exercise performance, the incremental load power cycling protocol used in this study effectively and safely induced participants to reach exhaustion, successfully establishing a model for endurance exercise. Furthermore, statistical analysis results indicated a significant prolongation in time to exhaustion for participants under the music condition compared to the no-music condition (*p* < 0.001), suggesting that music intervention significantly enhanced endurance exercise performance.

### 3.2. Differences in Brain Network Properties

Calculation of global network metrics was performed on the PLV matrix data of CEN, DMN, SN, SMN, and DAN using the Gretna toolbox, which included Eglob, Eloc, Cp, and Gamma (Table 2). The results of the two-way repeated-measures ANOVA for global network metrics in the Theta, Alpha, and Beta bands are shown in Table 3.

In terms of global network metrics in the Theta band, the results of a two-way repeated measures ANOVA indicated that the interaction effect of time and condition on Eglob was not significant [F_(1,33)_ = 1.364, *p* = 0.251, η^2^ = 0.040]. The main effect of time on Eglob was not significant [F_(1,33)_ = 1.099, *p* = 0.302, η^2^ = 0.032], while the main effect of condition was significant [F_(1,33)_ = 30.405, *p* < 0.001, η^2^ = 0.480]. Further paired comparisons (Bonferroni-corrected) revealed that Eglob was significantly higher with music compared to without music, both before and after exhaustive exercise (*p* < 0.01). Regarding Eloc, the interaction effect of time and condition was not significant [F_(1,33)_ = 0.156, *p* = 0.695, η^2^ = 0.005]. The main effect of time on Eloc was not significant [F_(1,33)_ = 2.876, *p* = 0.099, η^2^ = 0.080], while the main effect of condition was significant [F_(1,33)_ = 10.845, *p* = 0.002, η^2^ = 0.247]. Further paired comparisons indicated that Eloc was significantly higher with music compared to without music, both before and after exhaustive exercise (*p* < 0.01). For the Cp metric, the interaction effect of time and condition [F_(1,33)_ = 0.880, *p* = 0.355, η^2^ = 0.026], main effect of time [F_(1,33)_ = 0.637, *p* = 0.431, η^2^ = 0.019], and main effect of condition [F_(1,33)_ = 0.813, *p* = 0.374, η^2^ = 0.024] were all not significant. Regarding the Gamma metric, the interaction effect of time and condition was not statistically significant [F_(1,33)_ = 0.084, *p* = 0.774, η^2^ = 0.003]. The main effect of time on Gamma was not significant [F_(1,33)_ = 3.517, *p* = 0.070, η^2^ = 0.096], while the main effect of condition was significant [F_(1,33)_ = 7.881, *p* = 0.008, η^2^ = 0.193]. Further pairwise comparisons revealed that Gamma values were significantly higher with music compared to without music (*p* < 0.01).

In the global network indicators of the Alpha band, a two-way repeated measures analysis of variance showed that the interaction effect of time and condition on Eglob was not statistically significant [F_(1,33)_ = 0.014, *p* = 0.908, η^2^ = 0.000). The main effect of time was not significant [F_(1,33)_ = 0.050, *p* = 0.825, η^2^ = 0.001], while the main effect of condition was significant [F_(1,33)_ = 17.775, *p* < 0.001, η^2^ = 0.350]. Further pairwise comparisons revealed that Eglob significantly increased when music was present before exercise (*p* < 0.01) and was significantly higher after exercise with music compared to without music (*p* < 0.05). The interaction effect of time and condition on Eloc was not significant [F_(1,33)_ = 0.001, *p* = 0.980, η^2^ = 0.000]. The main effect of time was not significant [F_(1,33)_ = 1.843, *p* = 0.184, η^2^ = 0.053], while the main effect of condition was significant [F_(1,33)_ = 4.841, *p* = 0.035, η^2^ = 0.128]. Further pairwise comparisons showed that Eloc was significantly higher before exercise with music compared to without music (*p* < 0.05). For Cp, the interaction effect of time and condition [F_(1,33)_ = 1.273, *p* = 0.267, η^2^ = 0.037], main effect of time [F_(1,33)_ = 0.085, *p* = 0.773, η^2^ = 0.003], and main effect of condition [F_(1,33)_ = 0.009, *p* = 0.923, η^2^ = 0.000] were all not significant. Similarly, for Gamma, the interaction effect of time and condition [F_(1,33)_ = 0.141, *p* = 0.710, η^2^ = 0.004], main effect of time [F_(1,33)_ = 1.831, *p* = 0.183, η^2^ = 0.053], and main effect of condition [F_(1,33)_ = 0.724, *p* = 0.401, η^2^ = 0.021] were all not significant.

Analysis of global network indicators in the Beta frequency band revealed that the interaction effect of time and condition on Eglob was not statistically significant (F_(1,33)_ = 2.894, *p* = 0.098, η^2^ = 0.081). The main effect of time was not significant [F_(1,33)_ = 0.199, *p* = 0.659, η^2^ = 0.006), while the main effect of condition was significant [F_(1,33)_ = 24.730, *p* < 0.001, η^2^ = 0.428]. Further pairwise comparisons showed that Eglob was significantly higher with music before exercise (*p* < 0.05) and significantly higher with music after exercise (*p* < 0.001) compared to the no music condition. For Eloc, the interaction effect of time and condition was not statistically significant [F_(1,33)_ = 0.931, *p* = 0.342, η^2^ = 0.027]. The main effect of time was not significant [F_(1,33)_ = 2.857, *p* = 0.100, η^2^ = 0.080], while the main effect of condition was significant [F_(1,33)_ = 11.222, *p* = 0.002, η^2^ = 0.254]. Pairwise comparisons revealed that Eloc was higher with music conditions both before and after exercise compared to the no music condition (*p* < 0.05). No significant effects were found for Cp in terms of the interaction effect of time and condition [F_(1,33)_ = 0.334, *p* = 0.567, η^2^ = 0.010], the main effect of time [F_(1,33)_ = 1.052, *p* = 0.313, η^2^ = 0.031], and the main effect of condition [F_(1,33)_ = 1.195, *p* = 0.282, η^2^ = 0.035]. Similarly, for the Gamma indicator, the interaction effect of time and condition [F_(1,33)_ = 0.109, *p* = 0.743, η^2^ = 0.003], the main effect of time [F_(1,33)_ = 1.977, *p* = 0.3169, η^2^ = 0.057], and the main effect of condition [F_(1,33)_ = 3.052, *p* = 0.090, η^2^ = 0.085] were all not statistically significant.

### 3.3. Differences in Functional Connectivity of Brain Networks

Clean EEG data were imported into the Gretna software (version 2.0.0) for analysis of brain functional network connectivity. The study compared the total average PLV values of theta, alpha, and beta bands before and after movement under conditions with and without music. Significant differences in brain region connections were visualized using the BrainNet Viewer tool for edges with significant changes.

**Figure 3:**
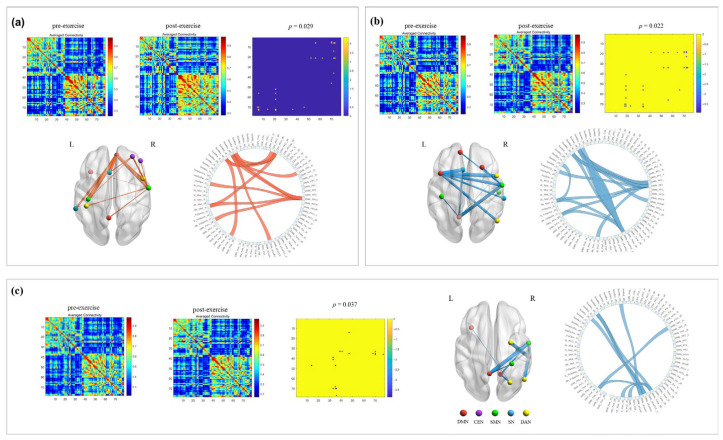
Frequency-specific network connectivity changes: pre- vs. post-exercise. Note: (**a**) beta-band network connectivity changes (without music); (**b**) alpha-band network connectivity changes (with music); (**c**) beta-band network connectivity changes (with music).

Comparison of pre- and post-exercise across frequency bands, results showed a significant increase in total average PLV values in the beta band after movement without music (*p* = 0.029). This enhancement was observed in strengthened connections in the right CEN, left SN, and DMN, and bilateral SMN and DAN, suggesting increased resource mobilization in the brain to maintain motor performance under fatigue (Figure 3a). Conversely, under music conditions, a significant decrease in total average PLV values in the alpha band after movement was found (*p* = 0.022). Weaker connections involved decreased connectivity in the right DAN and bilateral DMN, SMN, and SN, indicating that music may facilitate the brain transitioning to a state of higher arousal and more efficient information processing post-exercise (Figure 3b). In the beta band under music conditions, a significant decrease in total average PLV values after movement was observed (*p* = 0.037), with decreased connections in bilateral DMN, right SMN, and DAN (Figure 3c). This contrasts with the pattern seen without music, collectively demonstrating the positive role of music in optimizing efficiency in movement-related brain networks.

**Figure 4:**
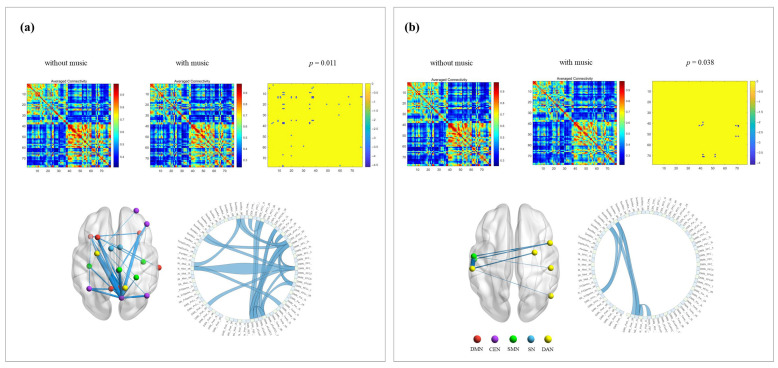
Frequency-specific network connectivity changes per-exercise: without music vs. with music. Note: (**a**) alpha-band network connectivity changes; (**b**) beta-band network connectivity changes.

**Figure 5:**
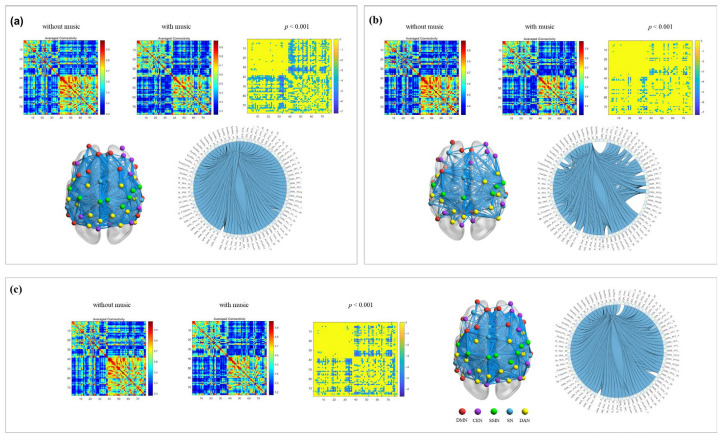
Frequency-specific network connectivity changes post-exercise: without music vs. with music. Note: (**a**) theta-band network connectivity changes; (**b**) alpha-band network connectivity changes; (**c**) beta-band network connectivity changes.

In comparison between the music and without music conditions in different frequency bands before exercise, as shown in Figure 4, the total average PLV value significantly decreased under the music condition compared to the without music condition (*p* = 0.011) in the Alpha band. Further network-level analysis revealed that the differences were mainly concentrated on changes in connectivity between nodes of the central DMN, CEN, and SMN, showing enhanced connections within these networks and between them. In the Beta band, the total average PLV value also significantly decreased under the music condition compared to the without music condition (*p* = 0.038), with this reduction mainly manifested as a decrease in connectivity between the left SMN and right DAN networks. After exercise, the PLV values in the delta, alpha, and beta frequency bands showed a significant decrease when compared between the conditions with and without music (*p* < 0.001), indicating a general weakening of connectivity strength across the whole brain (Figure 5).

### 3.4. Calculation of Functional Connectivity Strength Within Networks

**Figure 6:**
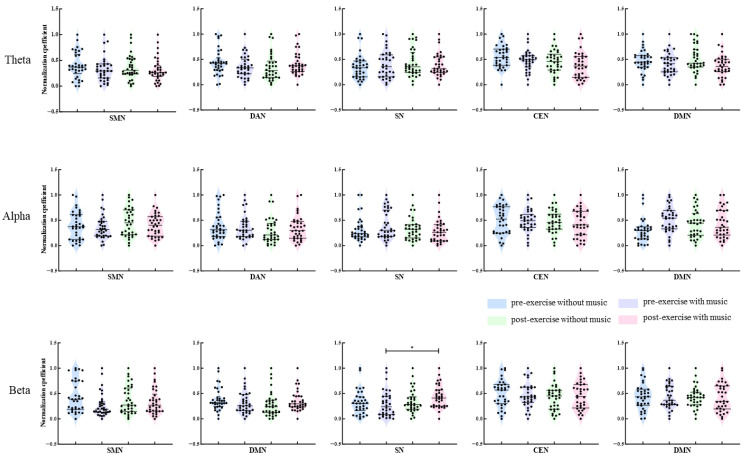
Comparison of connection strength characteristics across frequency bands and conditions. Note: *: *p* < 0.05.

Non-parametric tests were utilized to compare functional connectivity strengths within the SMN, DAN, SN, CEN, and DMN networks across Theta, Alpha, and Beta frequency bands. The Friedman test was employed for overall comparisons among the four conditions (without music before exercise, with music before exercise, without music after exercise, with music after exercise). Post-hoc pairwise comparisons were conducted using the Dunn multiple comparison test with significance levels adjusted through the Bonferroni method. As shown in Figure 6, the connectivity strength within the SN network significantly increased in the Beta frequency band after exhaustive exercise with music intervention compared to before exercise (*p* = 0.034). No significant differences were observed in connectivity strengths within the networks in other frequency bands across various conditions (*p* > 0.05).

### 3.5. Calculation of Functional Connectivity Strength Between Networks

**Figure 7:**
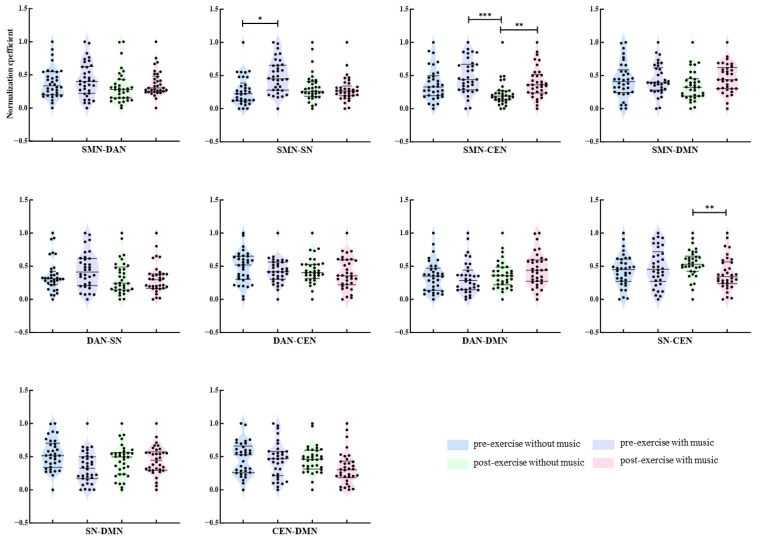
Between-network theta-band connectivity strength under different conditions. Note: *: *p* < 0.05; **: *p* < 0.01; ***: *p* < 0.001.

Non-parametric tests were utilized to compare the functional connectivity strength among the Theta, Alpha, and Beta bands of the SMN, DAN, SN, CEN, and DMN networks. The comparisons encompassed SMN-DAN, SMN-SN, SMN-CEN, SMN-DMN, DAN-SN, DAN-CEN, DAN-DMN, SN-CEN, SN-DMN, and CEN-DMN. The overall comparisons across the four conditions were carried out using the Friedman test. Subsequent pairwise comparisons were executed employing Dunn’s multiple comparison test with Bonferroni correction for significance levels. In the Theta band, depicted in Figure 7, prior to exhaustive exercise, the functional connectivity strength between the SMN-SN networks was notably higher under the music condition compared to the without music condition (*p* = 0.029). Concerning the SMN-CEN network connectivity strength, following exhaustive exercise, the strength under the music condition significantly surpassed the without music condition (*p* = 0.004); the connectivity strength between SMN-CEN networks before exercise with music was markedly greater than after exercise without music (*p* < 0.001). Furthermore, post-exhaustive exercise, the connectivity strength between SN-CEN networks under the music condition was significantly lower than under the without music condition (*p* = 0.001). No significant differences were observed in functional connectivity strength among the other network pairs under varying conditions (*p* > 0.05).

**Figure 8:**
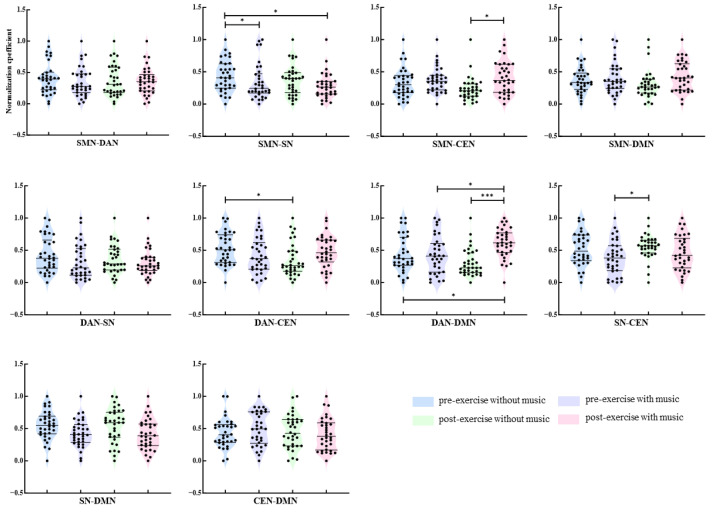
Between-network alpha-band connectivity strength under different conditions. Note: *: *p* < 0.05; ***: *p* < 0.001.

In the Alpha band (Figure 8), post-exhaustive exercise, the strength of connectivity between the SMN-CEN networks under music conditions was significantly higher than under without music conditions (*p* = 0.039). Prior to exercise, the functional connectivity strength between the SMN-SN networks under without music conditions was significantly higher than the connectivity strength under both music conditions before and after exercise (*p* = 0.039/0.016). Under without music conditions, post-exercise showed a significant decrease in functional connectivity strength between the DAN-CEN networks compared to pre-exercise (*p* = 0.029). The functional connectivity strength between the SN-CEN networks under without music conditions post-exercise was significantly higher than the connectivity strength under music conditions pre-exercise (*p* = 0.034). Regarding the DAN-DMN network connectivity strength, the study revealed that post-exercise, the connectivity strength under music conditions was significantly higher than under without music conditions (*p* < 0.001); when compared to pre-exercise under music conditions, post-exercise showed a significant increase in functional connectivity strength between the DAN-DMN networks (*p* = 0.039); the functional connectivity strength under music conditions post-exercise was significantly higher than under without music conditions pre-exercise (*p* = 0.039).

**Figure 9:**
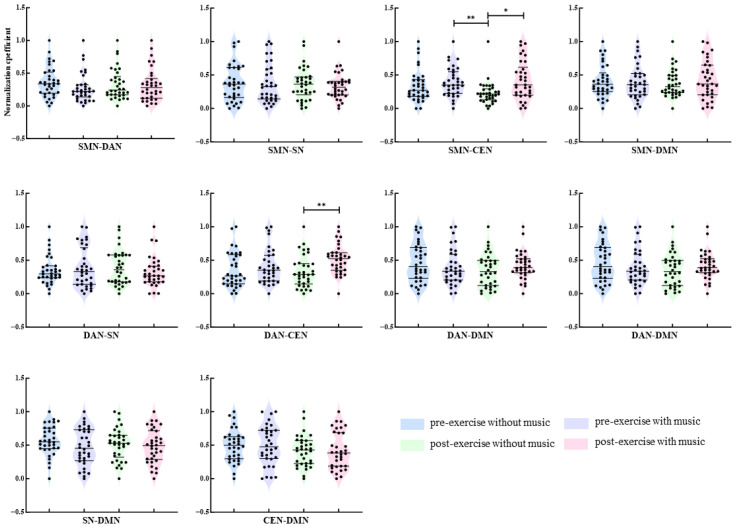
Between-network beta-band connectivity strength under different conditions. Note: *: *p* < 0.05; **: *p* < 0.01.

In the beta frequency band (Figure 9), post-exhaustion, there was a significant increase in functional connectivity strength between the DAN-CEN networks under the music condition compared to the without music condition (*p* = 0.010). After exercise, the functional connectivity strength between the SMN-CEN networks was significantly higher with music than without music (*p* = 0.034). Furthermore, the functional connectivity strength between the SMN-CEN networks under the music condition before exercise was significantly higher than under the without music condition after exercise (*p* = 0.007).

## 4. Discussion

This study combined high temporal resolution EEG technology with brain network analysis methods to systematically investigate the immediate effects of music on the resting-state brain functional connectivity of male athletes after exhaustive endurance exercise. The results not only reaffirmed the enhancing effect of music on endurance performance at the behavioral level but also revealed a unique pattern of music modulation on post-exercise brain functional networks at the neural level. The principal findings are threefold: (1) music intervention significantly prolonged the time to exhaustion, confirming its performance-enhancing effect; (2) music induced a state of higher global and local network efficiency across theta, alpha, and beta frequency bands, both before and after exercise; (3) exhaustive exercise without music led to a compensatory increase in beta-band connectivity, whereas music intervention prompted a decrease in alpha and beta-band connectivity post-exercise; and (4) music specifically modulated connectivity within and between key brain networks, notably enhancing the coupling between sensorimotor (SMN) and central executive (CEN) networks post-exhaustion. These results collectively suggest that music is associated with a more selectively integrated network configuration following exhaustive exercise. However, given the resting-state and post-exercise design, this pattern should be interpreted cautiously as being consistent with, rather than definitive evidence for, a neural efficiency mechanism underlying performance enhancement.

### 4.1. The Neural Efficiency Hypothesis for Music-Enhanced Exercise Performance

Research has shown that athletes significantly extend their time to exhaustion when listening to motivational music, aligning with previous studies on the ergogenic effects of music [5,6,32]. Furthermore, this study offers a neurophysiological explanation for this phenomenon through brain network analysis. The finding of this research is the significant main effect of the music condition on brain network efficiency. Specifically, we observed increased Eglob and Eloc in the theta, alpha, and beta bands when participants were exposed to music. Global efficiency reflects the overall capacity for information transfer across the entire network, while local efficiency indicates the robustness of information processing among neighboring nodes [28]. Thus, our results suggest that music primes the brain into a state of more efficient and integrated information processing, both at the whole-network level and within localized clusters of brain regions. This enhanced network configuration may be a key neural correlate of music’s psychological effects, such as improved mood, increased arousal, and attentional focus, which collectively contribute to improved physical performance.

The observed effects spanned multiple frequency bands, each potentially reflecting different aspects of music’s influence. The theta band is often implicated in emotional processing and cognitive control [33]. Increased theta network efficiency could signify a more effective regulation of the emotional and cognitive demands associated with anticipating and recovering from strenuous exercise. The alpha band is traditionally linked to a state of relaxed alertness [34] and the inhibition of irrelevant sensory information [35,36]. Enhanced alpha network efficiency might represent a more organized and functionally optimized “idling” state, allowing for better allocation of attentional resources and a reduced perception of distracting internal states like fatigue. The beta band is associated with active cognitive processing, sensorimotor function, and alertness [37]. Increased efficiency in beta-band networks could reflect a state of heightened readiness and more effective corticomuscular coupling, which is beneficial for motor task execution [38].

### 4.2. Brain Network Reorganization in Response to Fatigue

In the absence of music, exhaustive exercise induced a significant increase in total average connectivity in the beta frequency band. This heightened synchronization was widespread, involving connections within the CEN, SN, DMN, SMN, and DAN. Beta oscillations are critically involved in sensorimotor integration, attention, and maintaining the current cognitive-motor set [39,40]. The observed post-exercise increase in beta connectivity likely reflects a compensatory mechanism. As the central nervous system contends with fatigue signals, afferent feedback from muscles, and the need to maintain homeostasis, the brain may recruit additional neural resources and enhance communication across networks to preserve functional integrity and motor control [41]. This state of heightened connectivity can be interpreted as the neural signature of increased effort required to overcome the physiological stress induced by exhaustive exercise. This aligns with broader research on brain dynamics, which characterizes the brain as a complex system of functional networks that reconfigure in response to internal and external demands [42].

It is important to note that EEG recordings commenced within 30 s following exercise termination. Therefore, the observed connectivity patterns likely reflect a composite state including both central fatigue and acute physiological recovery dynamics (e.g., autonomic rebound, respiratory normalization, and arousal fluctuation). Consequently, the post-exercise connectivity differences between conditions should not be interpreted as exclusively reflecting fatigue-related neural mechanisms.

In stark contrast, when participants were exposed to music, exhaustive exercise led to a significant *decrease* in overall functional connectivity in both the alpha and beta bands. The reduction in alpha band connectivity is particularly telling, as increased alpha power is often associated with cortical idling or inhibition. A decrease in alpha connectivity post-exercise with music may therefore signify a transition to a state of higher arousal and more active, efficient information processing [43]. The concurrent decrease in beta band connectivity suggests that music enables the brain to achieve its post-exercise recovery state with less synchronous neural activity. Rather than the widespread, effortful hyper-connectivity seen without music, the brain under musical stimulation appears to operate more economically. This supports the notion that music can optimize the allocation of neural resources, a finding that resonates with studies showing music’s ability to alter functional connectivity in various contexts, such as in patients with major depressive disorder [44] and during emotional processing. Music may act as an external organizing stimulus, reducing the internal cognitive load associated with processing fatigue and interoceptive signals, thereby promoting a more efficient neural state.

### 4.3. Regulatory Effects of Music on Intra-Network and Inter-Network Functional Connectivity of the Brain

Beyond global changes, the most compelling evidence for music’s effect lies in the specific reorganization of communication between functional networks. The significantly enhanced functional connectivity between the SMN and CEN post-exercise under the music condition across theta, alpha, and beta bands. The SMN is responsible for processing sensory information and executing motor commands, while the CEN is crucial for top-down cognitive control, goal-directed behavior, and working memory [45]. The strengthened coupling between these two networks suggests that music facilitates a more robust integration of sensorimotor processes with executive functions during the recovery period [46]. This could translate to more efficient processing of bodily feedback (e.g., muscle soreness, heart rate) and better planning for subsequent actions, potentially accelerating recovery. This finding aligns with the therapeutic use of music in neurorehabilitation, where it is employed to enhance motor function by engaging multisensory and motor networks [47,48].

Furthermore, music modulated the interplay between attentional networks. Post-exercise, music increased the connectivity between the DAN and DMN in the alpha band. These two networks are typically anti-correlated, with the DAN engaged during externally focused tasks and the DMN active during internally focused thought or mind-wandering. The increased coupling observed here may indicate a more flexible and efficient switching of attention between interoceptive states (processed by DMN-related regions) and the external environment, guided by the auditory stream of music. This enhanced flexibility could be a mechanism by which music helps distract from the unpleasant sensations of fatigue.

The SN, which is critical for detecting and orienting attention towards relevant internal and external stimuli, also showed significant modulation [49]. The within-network connectivity of the SN increased in the beta band post-exercise with music. This suggests that music enhances the SN’s ability to process salient information, which in this context includes both the ongoing musical stimulus and the powerful interoceptive signals of exhaustion. This heightened SN activity could facilitate better emotional and autonomic regulation in response to physical stress, a core function of music’s therapeutic effect [50].

### 4.4. Research Limitations

The findings of this study hold significant theoretical and practical implications. Theoretically, it provides direct neurophysiological evidence for the enhancing effects of music, advancing our understanding of the “music-movement-brain” interaction to a new level of brain functional network dynamics from traditional psychological models like distraction and emotion regulation. The proposed “eural efficiency” model offers a unified neural framework for explaining how music can enhance performance and reduce subjective fatigue simultaneously. Practically, the results of this study offer solid theoretical support for the scientific application of music in sports training, competitive events, and clinical rehabilitation [8]. For instance, coaches and therapists can more effectively utilize music to optimize the neural states of athletes or patients, rather than just as a motivational tool. Future smart wearable devices may even be able to dynamically adjust music parameters based on real-time monitoring of brain signals to maintain the optimal “eural efficiency” state of the brain [51,52].

Although this study has made innovative findings, there are limitations that need to be addressed in future research. First, the sample of this study was limited to healthy young male sports science students, so the generalizability of the results to other populations (such as females, older adults, sedentary individuals, or clinical patients) remains to be validated. Considering potential gender differences in physiological responses to exercise [53], future studies should particularly include female participants. Second, although the use of personalized motivational music enhanced ecological validity and ensured strong motivational engagement, it also introduced inter-individual variability in musical characteristics, emotional responses, and familiarity. Music preference has been shown to play a critical role in modulating psychophysiological and neural responses to exercise [54,55]. Future research could systematically manipulate specific musical elements (e.g., tempo, rhythm, genre, lyrics, and volume) and compare standardized versus individualized music conditions to isolate their specific neural effects [56]. In addition, participants exercised for a longer duration under the music condition, although the same exhaustion criteria were applied in both conditions to ensure comparability of fatigue endpoints. However, the greater exercise load in the music condition may have contributed to the post-exercise neural differences observed. Future studies employing load-matched designs or statistical control based on covariates may help further isolate the specific neural effects of music. Third, although exercise performance improved significantly under the music condition, the present study did not directly examine correlations between behavioral performance variables (e.g., endurance time or cadence) and changes in functional connectivity metrics. As such, we cannot determine whether network alterations statistically mediate performance enhancement. Future studies should incorporate correlation and mediation analyses linking endurance performance to key graph metrics (e.g., Eglob, SMN–CEN coupling), and further examine whether such associations remain significant after controlling for recovery-related physiological proxies (e.g., heart rate at EEG onset or heart rate recovery slope). Future studies incorporating such correlation analyses, as well as continuous monitoring of physiological and perceptual responses (e.g., heart rate dynamics, rating of perceived exertion, and post-exercise affective or arousal states), may help further clarify the relationship between neural efficiency, fatigue perception, and performance enhancement. Fourth, EEG data were collected during resting-state periods before and after exercise rather than during exercise itself. While this approach minimizes motion artifacts and improves signal quality, it limits the ability to capture real-time neural dynamics during fatigue development. Future studies using mobile EEG systems or shorter post-exercise recording intervals may provide more detailed insights into the temporal evolution of brain network dynamics during exercise and recovery. Fifth, although EEG provides excellent temporal resolution, its spatial resolution remains limited compared with other neuroimaging modalities. Source reconstruction was performed using a standard template head model (MNI Colin27) and a three-shell boundary element model implemented in FieldTrip. The Schaefer100 atlas was registered to the template brain, and source activity was estimated using the LCMV beamformer. While template-based models are widely used in EEG research, the use of a standard head model and a relatively low channel count may introduce spatial uncertainty in source localization. Connectivity analysis was conducted at the source level, which helps reduce volume conduction effects compared with sensor-level analysis. Additionally, beamforming acts as a spatial filter that reduces signal leakage between regions. Nevertheless, PLV remains sensitive to residual field spread, which represents an inherent limitation of this connectivity metric. Sixth, this study adopted a cross-sectional design focusing on the acute effects of music during a single exercise session. Therefore, the present findings should be interpreted as reflecting phase synchronization patterns within methodological constraints, rather than definitive evidence of direct functional coupling. Future longitudinal studies are needed to determine whether long-term endurance training combined with music induces sustained changes in resting-state brain network organization and whether such changes are associated with long-term improvements in performance and fatigue resistance. Finally, the present study focused specifically on endurance exercise. Future research should extend this paradigm to other exercise modalities, such as resistance training [57,58], high-intensity interval exercise, and explosive movements, to examine the generalizability of the proposed neural efficiency model across different types of physical activity.

If the neural efficiency interpretation is valid, future studies should observe that connectivity efficiency metrics correlate positively with endurance performance, music-related reductions in beta hyper-connectivity predict lower perceived exertion, and such associations persist after controlling for recovery-related physiological indices. Testing these predictions will allow stronger mechanistic inference.

## 5. Conclusions

This study demonstrates that motivational music significantly modulates resting-state brain functional networks following exhaustive endurance exercise. While exercise without music induces widespread beta-band hyper-connectivity, reflecting increased neural cost under central fatigue, music promotes a more efficient and selectively integrated network configuration. Specifically, music enhances global network efficiency and facilitates adaptive coordination among sensorimotor, executive, and attentional networks. These findings are consistent with the neural efficiency hypothesis, although causal mechanisms cannot be established within the present design. Music may therefore serve as a practical and non-invasive strategy to enhance endurance performance and regulate central fatigue.

## Figures and Tables

**Table 1:** Effects of music on exhaustive endurance exercise performance (*n* = 34).

Music Condition	Exercise Termination Status				Exercise Duration (min)
	Maximum Heart Rate (beats/min)	RPE Rating	Behavioral Performance	Pedaling Cadence (rpm)	
Without Music	182.029 ± 2.099	19.500 ± 0.507	Dyspnea	<60	14.016 ± 2.562
With Music	181.941 ± 2.723	19.618 ± 0.493	Dyspnea	<60	17.191 ± 5.272 ***

Note: *** *p* < 0.001 compared to without music.

**Table 2:** Global network metrics for different frequency bands under different conditions.

Frequency Band	Metric	Without Music		With Music		*p*			
		Pre	Post	Pre	Post	Pre vs. Post		Without Music vs. With Music	
						Without Music	With Music	Per	Post
Theta	Eglob	0.231 ± 0.013	0.227 ± 0.016	0.241 ± 0.008	0.241 ± 0.009	0.215	1.000	0.001	<0.001
	Eloc	0.340 ± 0.012	0.336 ± 0.014	0.346 ± 0.007	0.343 ± 0.008	0.237	0.111	0.016	0.022
	Cp	0.287 ± 0.009	0.286 ± 0.012	0.286 ± 0.010	0.283 ± 0.008	0.889	0.151	0.892	0.221
	Gamma	0.815 ± 0.157	0.767 ± 0.142	0.869 ± 0.120	0.836 ± 0.123	0.194	0.294	0.113	0.057
Alpha	Eglob	0.226 ± 0.014	0.225 ± 0.016	0.235 ± 0.011	0.234 ± 0.013	0.007	0.016	0.846	0.947
	ELoc	0.337 ± 0.013	0.337 ± 0.014	0.343 ± 0.011	0.341 ± 0.011	0.374	0.229	0.044	0.202
	Cp	0.287 ± 0.010	0.288 ± 0.011	0.288 ± 0.010	0.286 ± 0.010	0.557	0.368	0.496	0.409
	Gamma	0.773 ± 0.145	0.785 ± 0.151	0.830 ± 0.156	0.795 ± 0.153	0.428	0.186	0.319	0.778
Beta	Eglob	0.231 ± 0.014	0.226 ± 0.018	0.238 ± 0.010	0.241 ± 0.009	0.229	0.243	0.015	<0.001
	ELoc	0.340 ± 0.010	0.335 ± 0.015	0.344 ± 0.006	0.342 ± 0.007	0.149	0.431	0.044	0.021
	Cp	0.286 ± 0.010	0.285 ± 0.012	0.285 ± 0.010	0.282 ± 0.007	0.746	0.214	0.677	0.223
	Gamma	0.814 ± 0.160	0.769 ± 0.158	0.840 ± 0.115	0.811 ± 0.118	0.297	0.329	0.319	0.262

**Table 3:** Results of two-way repeated-measures ANOVA for global network metrics across frequency bands.

Frequency Band	Metric	Main Effect of Time			Main Effect of Condition			Interaction Effect		
		F (*df*)	*p*	η^2^	F (*df*)	*p*	η^2^	F (*df*)	*p*	η^2^
Theta	Eglob	1.099 (1,33)	0.302	0.032	30.405 (1,33)	<0.001	0.480	1.364 (1,33)	0.251	0.040
	Eloc	2.876 (1,33)	0.099	0.080	10.845 (1,33)	0.002	0.247	0.156 (1,33)	0.695	0.005
	Cp	0.637 (1,33)	0.431	0.019	0.813 (1,33)	0.374	0.024	0.880 (1,33)	0.355	0.026
	Gamma	3.517 (1,33)	0.070	0.096	7.881 (1,33)	0.008	0.193	0.084 (1,33)	0.774	0.003
Alpha	Eglob	0.050 (1,33)	0.825	0.001	17.775 (1,33)	<0.001	0.350	0.014 (1,33)	0.908	0.000
	ELoc	1.843 (1,33)	0.184	0.053	4.841 (1,33)	0.035	0.128	0.001 (1,33)	0.980	0.000
	Cp	0.085 (1,33)	0.773	0.003	0.009 (1,33)	0.923	0.000	1.273 (1,33)	0.267	0.037
	Gamma	1.831 (1,33)	0.185	0.053	0.724 (1,33)	0.401	0.021	0.141 (1,33)	0.710	0.004
Beta	Eglob	0.199 (1,33)	0.659	0.006	24.730 (1,33)	<0.001	0.428	2.894 (1,33)	0.098	0.081
	ELoc	2.857 (1,33)	0.100	0.080	11.222 (1,33)	0.002	0.254	0.931 (1,33)	0.342	0.027
	Cp	1.052 (1,33)	0.313	0.031	1.195 (1,33)	0.282	0.035	0.334 (1,33)	0.567	0.010
	Gamma	1.977 (1,33)	0.169	0.057	3.052 (1,33)	0.090	0.085	0.109 (1,33)	0.743	0.003

## Data Availability

The original contributions presented in this study are included in the article/Supplementary Material. Further inquiries can be directed to the corresponding author.

## Supplementary Materials

The following supporting information can be downloaded at https://www.mdpi.com/article/10.3390/brainsci16030258/s1, Data.

## Abbreviations

The following abbreviations are used in this manuscript:

CNS	Central nervous system
RPE	Rated perceived exertion
EEG	Electroencephalogram
BMRI-2	Brunel Music Rating Inventory-2
ICA	Independent component analysis
CEN	Central executive network
DMN	Default mode network
SN	Salience network
SMN	Sensorimotor network
DNA	Dorsal attention network
PLV	Phase locking value
NBS	Network-based statistic
